# The Genetic Predisposition and Its Impact on the Diabetes Mellitus Development in Patients with Alcoholic Chronic Pancreatitis

**DOI:** 10.1155/2015/309156

**Published:** 2015-03-09

**Authors:** Agnieszka Madro, Marzanna Ciesielka, Krzysztof Celinski, Maria Slomka, Grazyna Czechowska, Jacek Kurzepa, Beata Kaszelan-Szczerbinska, Grzegorz Buszewicz, Roman Madro

**Affiliations:** ^1^Department of Gastroenterology with Endoscopic Units, Medical University, Jaczewski Street 8, 20-954 Lublin, Poland; ^2^Department of Forensic Medicine, Medical University, Ceramiczna Street 1, 20-150 Lublin, Poland; ^3^Department of Medical Chemistry, Medical University, Chodźki Street 4a, 20-093 Lublin, Poland

## Abstract

The most common cause of chronic pancreatitis (CP) is alcohol abuse. The aim of the present study was to identify patients with genetic predisposition to CP abusing alcohol. The question posed was whether CP manifests at a younger age and diabetes mellitus develops earlier in individuals with genetic predisposition. The study encompassed 79 patients with alcoholic chronic pancreatitis (ACP) and control group (100 persons). The following mutations were determined: R122H and N29I of PRSS1 and N34S of SPINK1 as well as E366K and E288V of SERPINA 1. No R122H and N291 mutations were observed in the group of ACP patients and in controls. Moreover, there was no E288V mutation. In 79 ACP patients, six SPINK 1 (N34S/wt) mutations were observed. In the control group, one heterozygous SPINK 1N34S gene mutation was found (*P* = 0.0238). Two PiZ mutations were identified in patients with ACP and one analogical mutation in controls. Amongst patients with ACP as well as SPINK1 and PiZ mutations, the onset of disease was observed earlier and developed earlier. The prevalence of SPINK1 mutation is higher in patients with ACP than in healthy populations. This mutation together with the effects of alcohol accelerates the development of ACP and of diabetes mellitus.

## 1. Introduction

Chronic pancreatitis (CP), irrespective of its aetiology, remains the disease for which modern medicine cannot offer effective therapeutic strategies. Special difficulties are encountered in cases with genetic predisposition underlying the condition. To date, many genes responsible for the development of CP have been identified. Genetic predisposition to CP was first described in 1952 [1]. Numerous mutations have been identified since then, among which mutations in the secretory trypsin inhibitor (SPINK 1) gene, cationic trypsinogen (PRSS1) gene, cystic fibrosis transmembrane conductance regular (CFTR) gene, chymotrypsinogen C (CTRC) gene, and calcium sensing receptor (CASR) gene [2–5] are considered to be essential. Nevertheless, the most common cause of CP in western countries is alcohol abuse. This aetiological factor concerns 38% of male and 11% of female patients [6]. Cigarette smoking is an independent risk factor of CP, dependent on the number of cigarettes smoked, which also contributes to increased severity of CP in cases of simultaneous alcohol abuse [6].

The mutations in SPINK 1 (including the N34S one) were first described by Witt in 2000. However, the prevalence of this mutation is quite high, that is, 0.5–2.5% in general population, which demonstrates that its presence is not sufficient for the development of CP. The mutation in question is rather suggested to be the additional modifying factor of CP [7].

Mutations in PRSS1 were discovered thanks to studies in individuals with hereditary CP. In 1996, Whitcomb described the sequence of five exons of PRSS1 and PRSS2 genes using genomic DNA from patients with hereditary pancreatitis (HP). To explain why the R122H mutation might cause pancreatitis, the researcher proposed that the Arg122-Val123 autolytic peptide bond in trypsin plays a crucial role in the degradation of prematurely activated trypsin in the pancreas [8]. Another important mutation is the N29I mutation, which, however, was found to have no effect on trypsin activity and trypsinogen stability by biochemical analysis using recombinant trypsinogen. The N29I mutation is hypothesized to change the higher-order structure of trypsin, resulting in decreased SPINK1 binding and increased autoactivation [9, 10]. In the following years, further mutations of PRSS1 were discovered in patients with hereditary or idiopathic CP [4]; nevertheless, R122H and N29I mutations are the most common PRSS1 mutations worldwide [11].

Recently, Rosendahl and coworkers published the largest study regarding CFTR, SPINK1, CTRC and PRSS1 genes in patients with idiopathic CP (ICP) and hereditary CP (HP). However, they excluded from the study patients abusing alcohol. According to their findings, a proportion of patients with the expression of PRSS1, SPINK1, and CTRC was markedly larger. The PRSS1 variants were mainly observed in younger CP patients [12].

With regard to alfa 1 antitrypsin (AAT), the genetic variants of this enzyme do not appear to play a predominant role in alcoholic chronic pancreatitis (ACP) [13]. However, little is known about its possible impact on the pathogenesis of CP. PiS and PiZ mutations are implicated yet the available studies have not demonstrated increased frequency of these mutations in patients with CP [14].

The aim of the present study was to identify individuals with genetic predisposition to CP amongst the patients treated for this condition with the history of alcohol abuse. The question posed was whether CP manifests at a younger age and diabetes mellitus develops earlier in individuals with genetic predisposition who abuse alcohol and smoke cigarettes.

## 2. Material and Methods

The study encompassed 79 patients with alcoholic chronic pancreatitis. The diagnosis was based on reliable criteria (fibrosis, calcifications in the pancreas) found on imaging examinations (abdominal CT, MRI, US) or histopathological results after surgery due to CP. The alcoholic aetiology was determined based on the history of alcohol abuse (>80 g/day for male and >60 g/day for female patients) for at least 5 years. The hereditary aetiology was also excluded based on history; none of the patients had a relative in the direct line diagnosed with CP. Moreover, hyperparathyroidism and hypertriglyceridemia were excluded as causes of the condition. Our patients have been consulted in the Outpatient Gastroenterology Clinic for at least 2 years. The demographic data were presented in Table 2. The control group included 100 randomly chosen unrelated individuals (50 women and 50 men) from south-eastern Poland. All control individuals declared only occasional alcohol consumption. Based on physical and imaging examinations CP was excluded in the control group. All patients gave their written informed consent for inclusion in the study. The study was carried in the Department of Gastroenterology with Endoscopic Unit, Medical University of Lublin in cooperation with the Department of Forensic Medicine and Department of Medical Chemistry. The study design was approved by the Bioethics Committee, Medical University of Lublin.

Blood for EDTA was sampled from individuals of the study and control group; subsequently, DNA was isolated using the QIAamp DNA Mini Kit (Qiagen). Quantitative and qualitative analysis of DNA was evaluated using the NonoDrop 1000 spectrometer (Thermo Scientific). The following mutations were identified: R122H (rs111033565) and N29I (rs111033566) in exon 3 and 2 of the PRSS1 gene, respectively, N34S (rs17107315) in exon 3 of the SPINK1 gene and E366K (rs28929474) in exon 5 as well as E288V (rs17580) in exon 3 of the SERPINA 1 gene. Determinations were conducted using the 7500 RT-PCR System (Applied Biosystems) with HID RT-PCR Analysis v.1.0 software according to the standard protocol. Mutations were examined using the following assays: assay ID: C_1157434_10, the N34S mutation, assay ID: C_34508510_10, the E342K mutation, assay ID: C_594695_20, the E288V mutation; in the case of R122H mutation, starters and allele-specific probes for wild-type and mutated variants were designed by Life Technologies. The N291 mutation was determined using the cyclic sequencing technique and the same primers as those used for the amplification reaction. The total volume of reaction mixture was 25 *μ*L and contained 2.5 *μ*L of 10x Taq DNA Polymerase Buffer (Promega), 10 pmol of each primer (Genomed), 1 U Tag DNA Polymerase (Promega), 0.2 mM of each dNTP (Promega) and 0.2 25–30 ng of DNA. Amplification was carried out in the T3 thermoblock (Biometra) under the following conditions: initial denaturation at 95°C for 2 min; 35 cycles of denaturation at 94°C for 30 sec, primer annealing at 64°C for 30 sec, extension at 72°C for 30 sec and final extension at 72°C for 7 min. The purified PCR products were sequenced using the BigDye Terminator v.3.1 Cycle Sequencing kit according to the standard protocol and the Thermoblock 9700 (Applied Biosystems). The products of sequencing were separated by capillary electrophoresis on the 3130 Genetic Analyzer. The results were analysed using SeqScape v.2.5 software. In addition, the presence of genetic variants was confirmed by direct sequencing using the BigDye terminator cycle sequence ready kit and the ABI 3130 Genetic Analyzer (Applied Biosystems). All the mutations identified in the control and study groups were confirmed by sequencing.  Table 1 presents sequences of primers and their hybridization temperatures in the reaction of DNA amplification and sequencing (Table 1).

## 3. Results

The vast majority of patients with ACP were men (65/79); the mean age of patients was 43.24 ± 12.67. Diabetes mellitus was diagnosed in 24/79 patients. A higher proportion of smokers were among ACP patients compared to controls (Table 2).

In the ACP and control groups, there were no PRSS1 mutations, both R122H and N291, or the PiS E288V mutation. Amongst 79 CP patients abusing alcohol, 4 N34S (het) mutations of the SPINK1 gene were found in men and 2 such mutations in women. In the control group (100 individuals), there was one N34S mutation of the SPINK1 gene. This difference was statistically significant (*P* = 0.0238). Moreover, two Piz mutations were observed in the ACP group and one analogical mutation in the control group, which was not statistically significant (Table 3).

In ACP patients with both the SPINK1 and PiZ mutation, the earlier onset of disease (36.72 ± 4.23 and 38.81 ± 5.28, resp.) and earlier development of diabetes mellitus (38.83 ± 3.21 and 39.75 ± 4.72, resp.) were observed. All results were not statistically significant. It was not possible to determine the impact of cigarette smoking due to too small groups of smokers and nonsmokers with the mutation and otherwise (Table 4).

## 4. Statistical Analysis

Statistical significance of differences in the distribution of SPINK1 mutations in the ACP and control group was checked using the fraction test (the two-sided test of structure coefficient). Since there were no grounds for rejection of a hypothesis for normal distribution and Fisher's (*F*) test did not demonstrate the significance of variance differences, differences in the average age of being affected and the average age of diabetes onset for groups with SPINK1 and ACP compared to controls, were assessed using Student's *t*-test (groups of small sizes).

Statistical analysis was performed using STATISTICA software (module of Descriptive Statistics for testing normality and Other Tests of Significance to calculate *P*).

## 5. Discussion

The most common cause of CP in western countries is undoubtedly alcohol abuse. Recent studies carried out in the United States, Italy and Denmark reveal that alcohol is the cause of CP in about 50% of cases [6]. In Brazil, alcohol is the cause of CP in 89.6% of cases [15]. Although alcohol drinking and cigarette smoking increase the risk of CP by 5 times, the disease was diagnosed only in 3% of alcoholics [16]. The above data suggest that some other factors contribute to the development of CP in alcohol abusers. Therefore, this group of patients was selected to our study. We attempted to determine whether the group contained individuals with genetic predisposition to CP and whether this might have affected the course of disease.

Interestingly, 5 genetic mutations listed earlier are rarer in alcoholics than it was believed. The data from 24 studies were analysed and published by Aoun et al. [17]; the most surprising was the fact that the incidence of SPINK1 mutations in alcohol abusers is low, which suggests that alcoholic CP develops via another pathway, independent of premature activation of trypsin [17]. In the alcoholic pathway, CP progression is most likely intensified by smoking and some genetic mutations, which induces direct activation of pancreatic stellate cells (PSC) and leads to fibrosis [5]. The above observations confirm our findings revealing the presence of SPINK1 mutations in 6 of 79 patients with CP. All the mutated individuals were heterozygote, which is contradictory to the results regarding Polish population published by Gasiorowska et al., who found 6 cases with the homozygous mutation [18].

In the Spanish population, similarly to other industrialised countries, alcohol is the main aetiological factor of CP. The N34S mutation in the SPINK1 gene was found in 3.9% of ACP patients, which is similar to the frequency (0–6%) observed in earlier studies in other European counties and in Japan [7, 19–21].

Chymotrypsin C (CTRC) is responsible for degradation of trypsin and trypsinogen isoforms while the mutations of CTPC gene account for the development of CP [22].

According to the study carried out by Rosendahl in 2008, the R254W mutation in exon 7 of the CTRC gene was present in 2.3% of ACP patients and in 0.5% of individuals amongst 432 patients with alcoholic chronic hepatic disease. Chang et al. identified some other variants and haplotypes of the CTRC gene in the population of 126 patients with CP of various aetiologies, whose frequency was only 2.3% [23].

The North American Pancreatic Study Group is currently carrying out research on genetic predisposition in alcoholics. The preliminary findings suggest the correlation between ACP and a locus on the X chromosome [5]. These extremely interesting results partially explain why males are heavy drinkers more often than females. In females, the high-risk allele acts as a recessive genetic disorder. The vast majority of our patients were male, which indirectly confirms that this gender is more susceptible to ACP.

Considering that other less known mutations can play some role in patients with ACP, we performed the determinations of PiZ and PiS mutations of the apha-1 antitrypsin (AAT) gene. Only two PiZ mutations were found in 79 ACP patients, confirming that this mutation is not essential for the development of ACP.

The study conducted by Mora et al. in the Spanish population demonstrated one PiZ mutation in a patient with ACP, which constituted 1.3%; in the groups of patients with idiopathic chronic pancreatitis (ICP) and HP, no such a mutation was observed [14]. The same frequency of this mutation was demonstrated in earlier studies in controls of the same population. Similar results were published by Witt et al.; that is, no differences in the PiS mutation frequency between the control group and patients with CP [24]. The PiS mutation frequency in CP patients in Spain (22%) was higher than that reported in Germany (4.1%) while the PiZ frequency was lower (0.96 versus 2%) [25, 26].

Another interesting issue is whether the presence of any mutation in patients with ACP accelerates the development of disease, including the occurrence of diabetes mellitus. In our patients with SPINK 1 and PiZ mutations, the onset of disease was earlier (in younger patients and in shorter time) compared to the remaining patients; moreover, diabetes mellitus also developed in younger patients and all the mutated were affected. However, it is not known whether the action of alcohol or the SPINK1 N34S mutation itself is crucial. According to Sun examining the Chinese population, patients with the c 194+2T>C SPINK1 mutation were diagnosed with the disease at younger age and developed diabetes earlier [27]. Noteworthy, he studied the population with idiopathic CP and did not find any N34S mutation, which is the variant more common in western countries [28–30].

The findings described above disclose that the SPINK 1 mutation is more commonly found in ACP patients compared to the healthy population and that its frequency is comparable to that observed in the group of CP patients [12]. The mutation in question together with the effects of alcohol probably accelerates the development of CP with mutations and the onset of diabetes mellitus, although two different mechanisms are likely to be involved. However, further studies in large populations are needed to confirm these observations.

## Figures and Tables

**Table 1:** Primer sequence and conditions for polymerase chain reaction-sequencing.

Variant	Primer sequence (forward and reverse)	Annealing (°C)

PRSS1 p.R122H	5′-GGT CCT GGG TCT CAT ACC TT-3′ 5′-GTA ATG GGC ACT CGA AAT GT-3′	60°

PRSS1 p.N29I	5′-CGC CAC CCC TAA CAT GCT AT-3′ 5′-CTC TCC CAG GCA GAC TGG CC-3′	64°

SPINK1 p.N34S	5′-CCA TTT CAG AGA TTT TGC TAT G-3′ 5′-GGT GAG ATT CAT ATT ATC AGT A-3′	60°

SERPINA1p. E366K	5′-AGC CTT ACA ACG TGT CTC TGC-3′ 5′-GGA TTT ACA GAT CAC ATG CAG G-3′	68°

SERPINA1p.E288V	5′-TCT TCC AAA CCT TCA CTC ACC-3′ 5′-GTC CCA ACA TGG CTA AGA GG-3′	61°

**Table 2:** Characteristics of groups.

Groups	Number of patients	Women	Men	Age (years)	Diabetes	Smoking
ACP	79	15	64	43.24 ± 12.67	24	46
Control	100	50	50	45.38 ± 10.21	0	23

ACP: alcoholic chronic pancreatitis.

**Table 3:** Distribution of PRSS1, SPINK1, PiZ, and PiS in ACP patients and controls.

	ACP	Control	*P* value
PRSS1	0/79	0/100	NS
R122H	0/79	0/100	NS
N29I	0/79	0/100	NS
SPINK1 N34S (het)	6/79	1/100	*P* = 0.0238
PiZ E342K	2/79	1/100	NS
PiS E288V	0/79	0/100	NS

ACP: alcoholic chronic pancreatitis.

**Table 4:** Clinical and genetic characteristics of patients with ACP.

	SPINK1	PiS	PiZ	PRSS1	ACP without mutation	*P* value
Women	2/15	0/15	0/15	0/15	13	**—**

Men	4/64	0/64	2/64	0/64	60	**—**

Age of onset	36.72 ± 4.23	—	38.81 ± 5.28	—	41.04 ± 6.51 41.04 ± 6.51	*P* = 0.1153 *P* = 0.6333

Diabetes	38.83 ± 3.21	—	39.75 ± 4.72	—	43.62 ± 6.73 43.62 ± 6.73	*P* = 0.0895 *P* = 0.4234

Smokers	2/6	—	0/2	—	46/71	**—**

ACP: alcoholic chronic pancreatitis.
